# Yam genetic diversity in Sub-Saharan Africa: implications for conservation and breeding

**DOI:** 10.1186/s12870-025-07935-3

**Published:** 2026-01-03

**Authors:** Amal Messadia, Paterne Agre, Kwabena Darkwa, Emmanuel Amponsah Adjei, Konan Evrard Brice Dibi, Lassana Bakayoko, Désiré N’Da Pokou, Alexandre Dansi, Amani Kouakou Michel, Idris Adejumobi, Adeyinka Adewumi, Jude Obidiegwu, Hapson Mushoriwa, Balogun Morufat, Amudalat Bolanle Olaniyan, Asrat Asfaw

**Affiliations:** 1 Pan African University Life and Earth Sciences Institute (Including Health and Agriculture) (PAULESI), University of Ibadan, Ibadan, Nigeria; 2 International Institute of Tropical Agriculture, Ibadan, Nigeria; 3 CSIR Savanna Agricultural Research Institute, Tamale, Ghana; 4 Station de Recherche sur les Cultures Vivrières (SRCV), Centre National de Recherche Agronomique (CNRA), Bouaké 633, Côte d’Ivoire; 5 Laboratoire Central de Biotechnologie (LCB), Centre National de Recherche Agronomique (CNRA), Abidjan, 1740 Côte d’Ivoire; 6 National High School of Applied Biosciences and Biotechnologies (ENSBBA), National University of Sciences, Technologies, Engineering and Mathematics (UNSTIM), Dassa-Zoumé, Republic of Benin; 7 National Root Crops Research Institute, Umudike, Abia State Nigeria; 8 Department of Crop and Horticultural Sciences, University of Ibadan, Ibadan, Nigeria

**Keywords:** Breeding, Genetic diversity, SNP markers, Sub-Saharan africa, Population structure

## Abstract

Yam (*Dioscorea* spp.) encompasses diverse species, including several staple food crops, of which a few were domesticated on the African continent. This study assessed yam genetic diversity in Sub-Saharan Africa (SSA) to inform breeding and conservation initiatives. A diverse collection of 1,247 yam accessions representing six species (*D. rotundata*,* D. alata*,* D. praehensilis*,* D. bulbifera*,* D. cayenensis*, and *D. dumetorum*) sourced from six African countries (Benin, Côte d’Ivoire, Democratic Republic of Congo, Ghana, Nigeria, and Uganda) was used in this study. Genetic diversity was assessed using 7,648 single-nucleotide polymorphism (SNP) markers, selected from previously sequenced datasets between the Consultative Group on International Agricultural Research (CGIAR) and National Agricultural Research and Extension Systems (NARES) collaboration. Findings showed a substantial inter- and intra-specific variation in African yam germplasm, with observed heterozygosity ranging from 0.165 to 0.464 and an average polymorphic information content (PIC) of 0.324 across populations. Population structure was assessed using ADMIXTURE (with cross-validation error for optimal K), DAPC (with BIC for K), and an IBS-based Neighbor-Joining (NJ) tree. Analysis of molecular variance (AMOVA) indicated moderate differentiation among countries (FST = 0.07), and higher differentiation among species (average FST = 0.14). Clustering patterns and phylogenetic analysis revealed the presence of evolutionary relationships among *D. cayenensis*,* D. praehensilis*, and *D. rotundata*, providing insights into *D. rotundata* domestication history in West Africa. These findings enhance our understanding of genetic relationships within the *Dioscorea* genus.

## Introduction

Yam (*Dioscorea* spp.) is a key food security crop in sub-Saharan Africa (SSA), particularly in West and central Africa, where it provides a reliable source of calories, income, and cultural identity for millions of rural households [1, 2]. In West Africa alone, over 300 million people rely on yam as a staple food [3]. In addition to its high carbohydrate content, yam contributes essential micronutrients and is integral to local ceremonies, festivals, and economies [4–6]. The region produces more than 90% of global yam output, making it the epicenter of yam cultivation [7, 8].

The genus *Dioscorea* comprises approximately 600 species, of which only 11 are cultivated for food or medicinal use [9–11]. Among these, *Dioscorea rotundata* (white yam) and *D. alata* (water yam) are the most widely grown, accounting for about 90% of global production. *D. rotundata* is indigenous to African, likely domesticated in West Africa’s Niger River basin from natural hybridization between the savannah species *D. abyssinica* and the rainforest species *D. praehensilis*, as evidenced by recent genomic studies [12, 13]. The yam domestication is still an ongoing process in Africa and offers valuable insights into how farmers harness genetic resources to cultivate crops that meet their agricultural needs [14]. This long domestication history underscores the region’s yam genetic diversity and its agronomic significance in the food system.

Genetic diversity in yam has been explored using both morphological and molecular tools. Studies have reported high diversity in Chinese yam (*D. opposita*) [15, 16] and regional differentiation in African species [17, 18]. For example, *D. rotundata* accessions in Benin show low to moderate diversity, shaped by seed exchange and clonal propagation [19]. Ethiopian yams have been shown to be genetically distinct from West African genotypes [20], suggesting independent evolutionary histories. Furthermore, local adaptation has driven within-country diversity in traits like flowering, tuber morphology, and yield [18].

Advances in molecular markers have enhanced the resolution of diversity studies. Simple Sequence Repeats (SSRs) and Amplified Fragment Length Polymorphism (AFLP) markers initially revealed high polymorphism [21, 22], while newer tools such as DArTseq and genotyping-by-sequencing (GBS) have facilitated genome-wide analysis. DArTseq has identified over 10,000 SNPs in *D. rotundata*, enabling structure and association mapping [10, 18, 23]. A 600 K SNP panel has also been used to reconstruct evolutionary relationships between wild and cultivated yams, revealing selection sweeps associated with domestication traits [12].

Despite these advances, major knowledge gaps persist in yam genetic research including geographical biases in research focus, limitations in the types of germplasm utilized, constraints in the application of molecular markers, and various bottlenecks in breeding programs [24–27].

Recent studies show a shift from reliance on genebank materials to farmer-managed landraces, emphasizing on-farm diversity and indigenous knowledge. For instance [28], collected and characterized yam cultivars from Ekiti State, Nigeria, while [29] analyzed chromosomal variation among cultivated species from southwest Nigeria. Similar efforts in Ethiopia documented folk taxonomy and morphological diversity of landraces [30, 31]. These studies highlight the value of locally adapted germplasm but remain geographically limited and lack genomic integration across regions. Thus, despite these advances, significant knowledge gaps persist in understanding the continent-wide genetic diversity and evolutionary relationships among *Dioscorea* spp. In countries like Kenya, the Democratic Republic of the Congo (DRC), yam remains poorly characterized or ignored entirely in genomic initiatives [32, 33]. These gaps hinder the identification of valuable alleles for stress tolerance, disease resistance, and nutritional traits. The continued use of low-resolution markers in some regions further limits the discovery of fine-scale genomic variation [22, 32].

Breeding programs are further challenged by the yam’s complex biology, which includes erratic flowering, poor seed set, dioecy, and heterozygosity [34]. Without a comprehensive understanding of available genetic variation across the continent, breeders risk narrowing the genetic base and missing opportunities to improve the crop’s resilience and productivity [35].

To address these gaps, this study assessed the genetic diversity and population structure of 1,247 yam accessions representing six *Dioscorea* species from six African countries: Uganda, Ghana, Nigeria, Benin, Côte d’Ivoire, and the Democratic Republic of Congo. Specifically, we aimed to evaluate the extent of genetic variation within and among yam species, assess the population structure and gene flow across geographic regions using high-density SNP markers.

## Materials and methods

### Plant materials

**Fig. 1:**
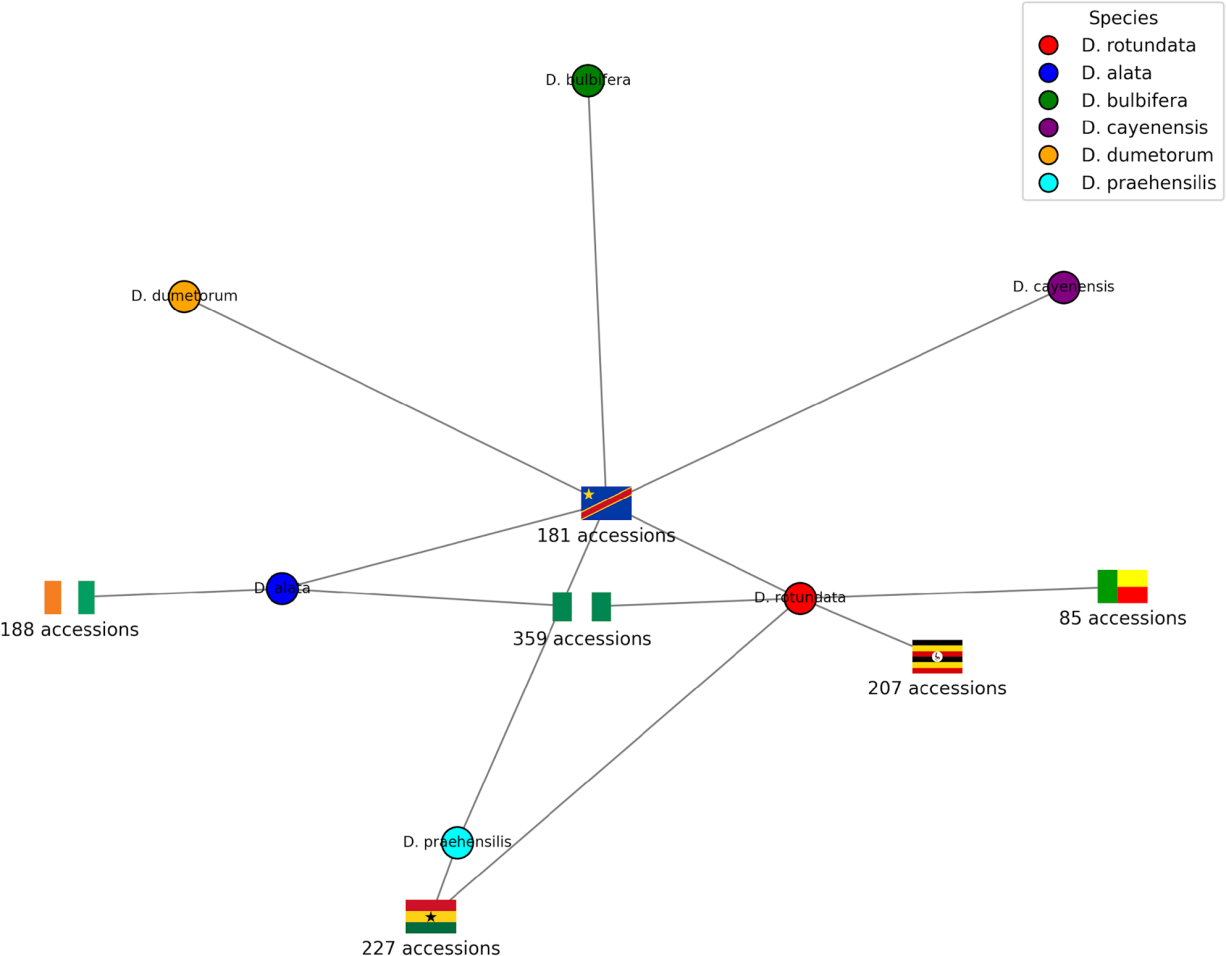
Origins of the 1247 accessions used in this study

A total of 1,247 yam genotypes from various published studies were selected from six countries and used in this study. Diversity Arrays Technology (DArT) sequencing was applied to generate six datasets used in this study. The first dataset comprised 207 *D. rotundata* accessions from Uganda [18]. In addition, 188 *D. alata* samples obtained from Côte d’Ivoire formed the second dataset [36]. The third dataset consisted of 227 *D. praehensilis* accessions collected from Ghana [37]. The fourth dataset was sourced from Benin that composed of 85 *D. rotundata* accessions [19]. The fifth dataset is included 194 accessions of both *D. rotundata* and *D. alata* from Nigeria [10, 35], and another 173 *D. rotundata* accessions generated through whole-genome sequencing [24]. The sixth dataset, composed of 182 accessions from DRC [38] (Fig. 1). All the data used in this study can be downloaded from the following link (https://figshare.com/account/items/29314031/edit**).** During the original germplasm collection, local names and origins of the yams were documented. Wild-related yam species collected from Ghana and the Democratic Republic of Congo were identified with the assistance of local farming communities and based on established descriptors, as reported in [39] and [38]. Farmers provided their consentbefore the collection, and the purpose of the collection was clearly explained to them.

## Dataset compilation and quality control

All variant call format (VCF) files generated from the above projects were merged using a customized Perl script, retaining only the common SNP marker positions to produce a unified VCF file containing all genotypes. Quality control involved merging VCF files for every dataset into a single VCF file. Filtering was done to remove SNP markers with poor quality using PLINK 1.9 and VCF tools. Markers with high missing values > 80% as well as duplicate SNPmarkers were removed. Markers with low minor allele frequencies (< 0.05) were all removed.

## Genotypic data analysis

Observed and expected heterozygosity, minor allele frequency (MAF), and polymorphic information content (PIC) were calculated with VCF tools and PLINK 1.9 [40]. CMplot package was also used for marker density and distribution on the 20 yam chromosomes [15]. SNP data were subjected to population structure analysis following the method described by [35]. By testing cluster numbers ranging from 2 to 50, the optimal number of clusters was identified through k-means analysis, employing cross-validation on the basis of the Bayesian information criterion (BIC). Each yam genotype was then assigned to its respective cluster if it had at least 70% ancestry probability. Genotypes with less than 70% ancestry were considered as admixed. The diversity pattern revealed through population structure analysis was further supported by discriminant analysis of the principal component (DAPC) via the Adegenet package [41]. Pairwise genetic dissimilarity distances based on identity-by-state (IBS) were estimated using plink1.9 version Linux command based. Using the IBS, hierarchical cluster dendrogram was generated and visualized using Phylogenetics and Evolution (APE) package in R [42]. Network analysis was conducted using NetworkX package in Python [43] with countries and yam species considered as factors. Analysis of molecular variance (AMOVA) within and among different yam species and countries was estimated using the fixation index (Fst) implemented in VCFtools.

## Results

### Genotypic summary statistics

**Fig. 2:**
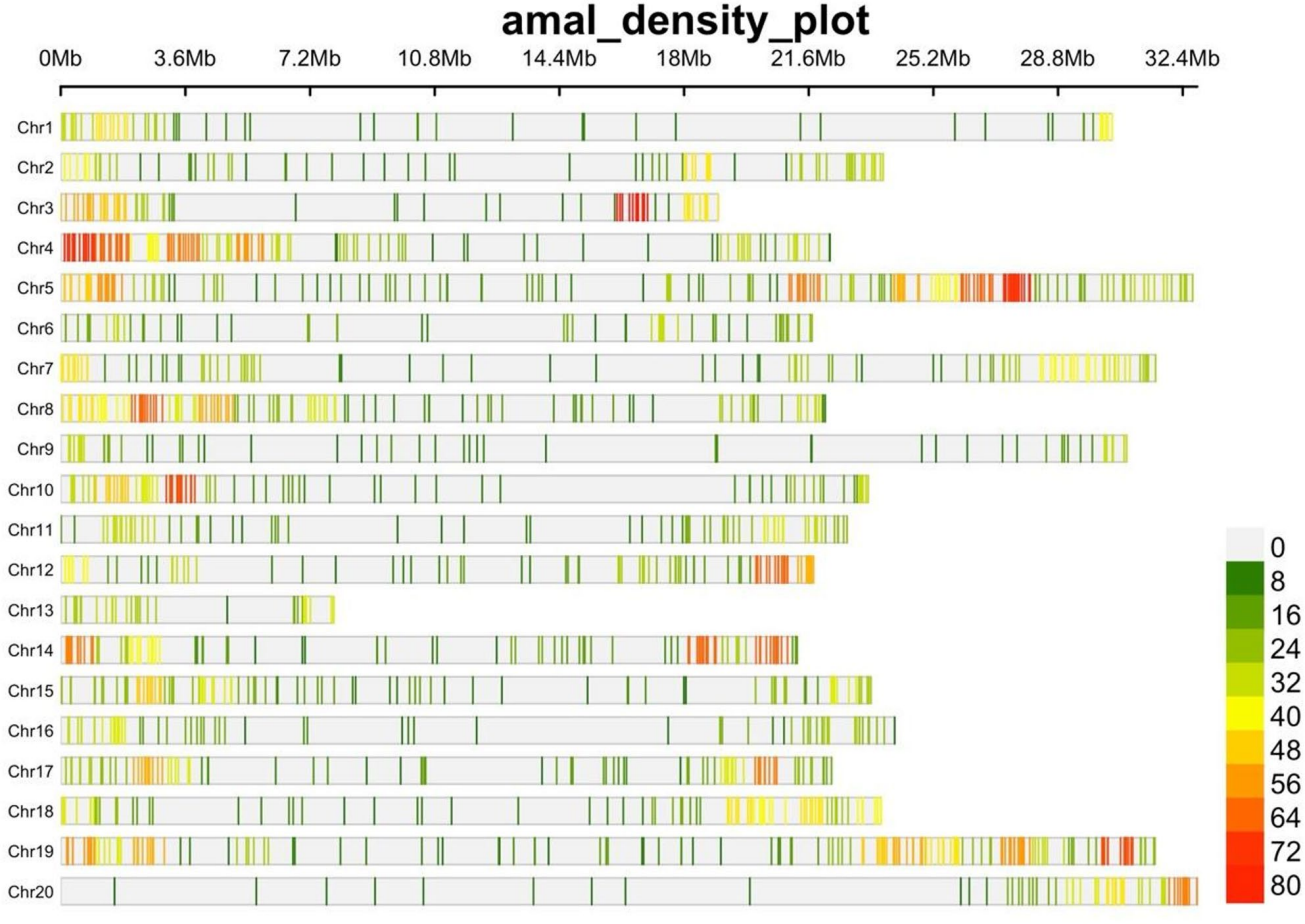
Distribution of SNP markers across the 20 yam chromosomes. Each color represents the marker density at a particular region of the yam chromosome

A total of 7,648 SNP markers were retained after filtering, with unequal distribution across the 20 yam chromosomes (Fig. 2; Table 1). The genome-wide SNP density plot indicated that chromosome 5 had the highest concentration of SNPs, accounting for 10.66% of the total number of markers with 816 SNPs. In contrast, chromosome 13 had the lowest marker concentration, with only 1.93% of the SNPs, totaling 148 markers. The diversity indices for the SNP markers presented a mean PIC value of 0.232, ranging from 0.194 on chromosome 15 to 0.265 on chromosome 6. The MAF averaged 0.213 across all the markers, with values ranging from 0.176 (chromosome 15) to 0.247 (chromosome 9). The observed heterozygosity (Ho) ranged from 0.209 on chromosome 3 to 0.337 on chromosome 6, with an average of 0.259. The expected heterozygosity (He) varied between 0.239 (chromosome 15) and 0.328 (chromosome 6), with an average of 0.288 (Table 1; Fig. 2).

**Table 1:** Summary statistics of SNP markers across 20 Yam chromosomes

Chromosome	No of SNPs	He	Ho	MAF	PIC
Chr1	256	0.262	0.239	0.182	0.216
Chr2	328	0.299	0.262	0.224	0.239
Chr3	312	0.272	0.209	0.196	0.222
Chr4	540	0.290	0.254	0.212	0.235
Chr5	816	0.266	0.236	0.200	0.214
Chr6	244	0.328	0.337	0.238	0.265
Chr7	436	0.284	0.262	0.200	0.232
Chr8	496	0.295	0.268	0.223	0.237
Chr9	228	0.327	0.292	0.247	0.260
Chr10	364	0.312	0.282	0.233	0.250
Chr11	296	0.292	0.280	0.219	0.234
Chr12	368	0.307	0.275	0.228	0.246
Chr13	148	0.306	0.263	0.237	0.243
Chr14	428	0.284	0.258	0.208	0.230
Chr15	376	0.239	0.219	0.176	0.194
Chr16	268	0.307	0.248	0.226	0.248
Chr17	408	0.281	0.255	0.215	0.225
Chr18	372	0.250	0.234	0.179	0.205
Chr19	700	0.286	0.266	0.211	0.232
Chr20	264	0.268	0.237	0.201	0.216
Total	7648				
average	382.4	0.288	0.259	0.213	0.232
SD		0.0231	0.0273	0.0196	0.0173

*Chr* chromosome, *Ho* observed heterozygosity *He* expected heterozygosity, *MAF* minor allele frequency, *PIC* polymorphic information content, *SD* standard Deviation

**Fig. 3:**
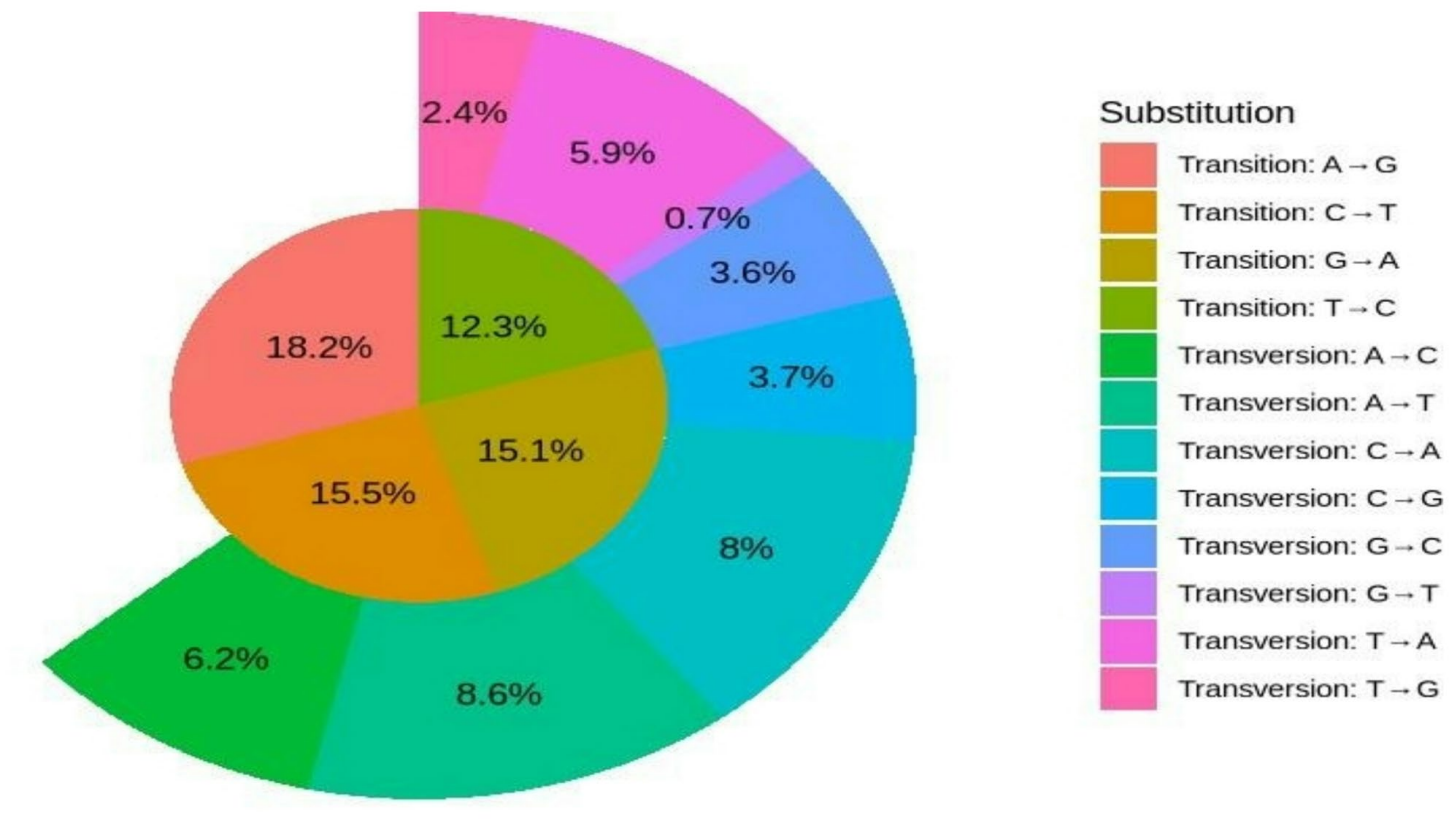
Rate of Transition and Transversion mutations based on bi-allelic SNP markers

In terms of mutation types, transition SNPs (Ts) accounted for 61.08% of the total SNPs, while transversion SNPs (Tv) represented 39.1% (Fig. 3). Among these, A/G transitions were the most common (18.2%), whereas G/T transversions were the least frequent (0.7%).

The genetic diversity parameters of yam accessions across six countries revealed moderately high levels of expected heterozygosity (He), ranging from 0.436 (Côte d’Ivoire) to 0.451 (Benin), with an overall average of 0.444. Observed heterozygosity (Ho) was slightly lower across all countries, with values ranging from 0.249 to 0.264. Nigeria exhibited the highest MAF (0.246) and PIC (0.302) while Uganda showed the lowest MAF (0.229) and PIC (0.289). Overall, findings indicate a relatively balanced level of genetic diversity across countries, with Nigeria slightly standing out for its higher diversity indices (Table 2).

**Table 2:** Summary of genetic diversity parameters (Ho, He, MAF, and PIC) in Yam accessions from six West, Central, and East African countries

Country	Ho	He	MAF	PIC
Benin	0.249	0.451	0.237	0.296
CIV	0.264	0.436	0.236	0.295
DRC	0.255	0.445	0.239	0.297
Ghana	0.253	0.447	0.235	0.295
Nigeria	0.258	0.442	0.246	0.302
Uganda	0.255	0.445	0.229	0.289
Average	0.256	0.444	0.237	0.296
SD	0.005	0.005	0.0051	0.0038

*SD* standard deviation, *Ho* observed heterozygosity *He* expected heterozygosity, *MAF* minor allele frequency, *PIC* polymorphism information content, *CIV* Cote d’Ivoire, *DRC* Democratic Republic of Congo

At the species level, genetic diversity analysis across six *Dioscorea* species revealed moderate to high expected heterozygosity (He), with values ranging from 0.412 in *D. bulbifera* to 0.454 in *D. cayenensis*. Observed heterozygosity (Ho) varied slightly among species, with *D. bulbifera* showing the highest Ho (0.288), suggesting relatively higher outcrossing or genetic variation in that species. Minor allele frequency (MAF) was consistent across species (average = 0.236), indicating comparable allelic distributions. Polymorphic information content (PIC) values ranged from 0.354 to 0.363. Overall, *D. rotundata*,* D. praehensilis*, and *D. dumetorum* exhibited slightly higher marker informativeness, supporting their relevance in breeding and conservation efforts (Table 3).

**Table 3:** Summary of genetic diversity parameters(Ho, He, MAF, and PIC) across six Yam species

Species	Ho	He	MAF	PIC
D. bulbifera	0.288	0.412	0.237	0.360
D. alata	0.266	0.434	0.230	0.354
D. cayenensis	0.246	0.454	0.237	0.360
D. dumetorum	0.255	0.445	0.237	0.362
D. praehensilis	0.253	0.447	0.236	0.361
D. rotundata	0.254	0.446	0.238	0.363
Average	0.259	0.441	0.236	0.360
SD	0.0137	0.0137	0.0027	0.0029

*Ho* observed heterozygosity, *He* expected heterozygosity, *MAF* minor allele frequency, *PIC* polymorphism information content

### Population stratification and diversity assessment

**Fig. 4:**
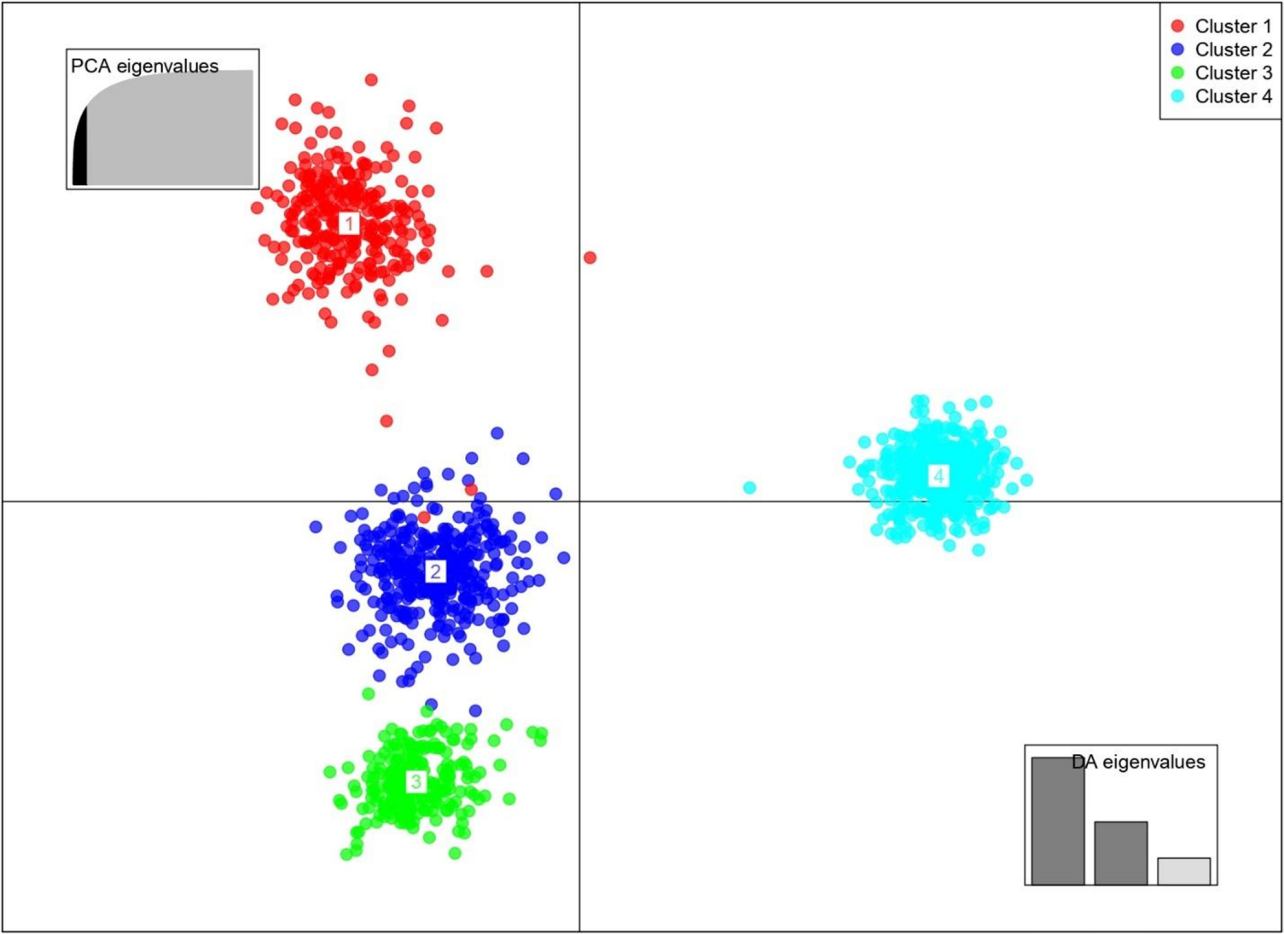
Discriminant analysis of principal components (DAPC) using 7648 SNP markers. Each color represents a cluster (cluster1 is red, cluster 2 is blue, cluster 3 is green, cluster 4 is cyan, and each dot represents an individual. Numbers represent the different subpopulations identified by DAPC analysis

Population structure was assessed using ADMIXTURE (with cross-validation error for optimal K), DAPC (with BIC for K), and an IBS-based Neighbor-Joining (NJ) tree, were employed to investigate the population structure of 1,247 yam accessions. Using the Bayesian Information Criteria (BIC), we notice K = 4 a rapid decline was observed suggesting the grouping of the entire population in 4 major groups (Fig. 4). Group 1 (red) included 180 accessions, primarily 165 of *Dioscorea praehensilis* from Ghana, with some from DRC and Uganda. Group 2 (blue) was made of 542 genotypes, mainly 524 *D. rotundata*. Group 3 (green) contained 194 accessions, mostly *D. praehensilis* and Group 4 (cyan) had 332 accessions made mainly *D. alata* (Figure. 4).

**Fig. 5:**
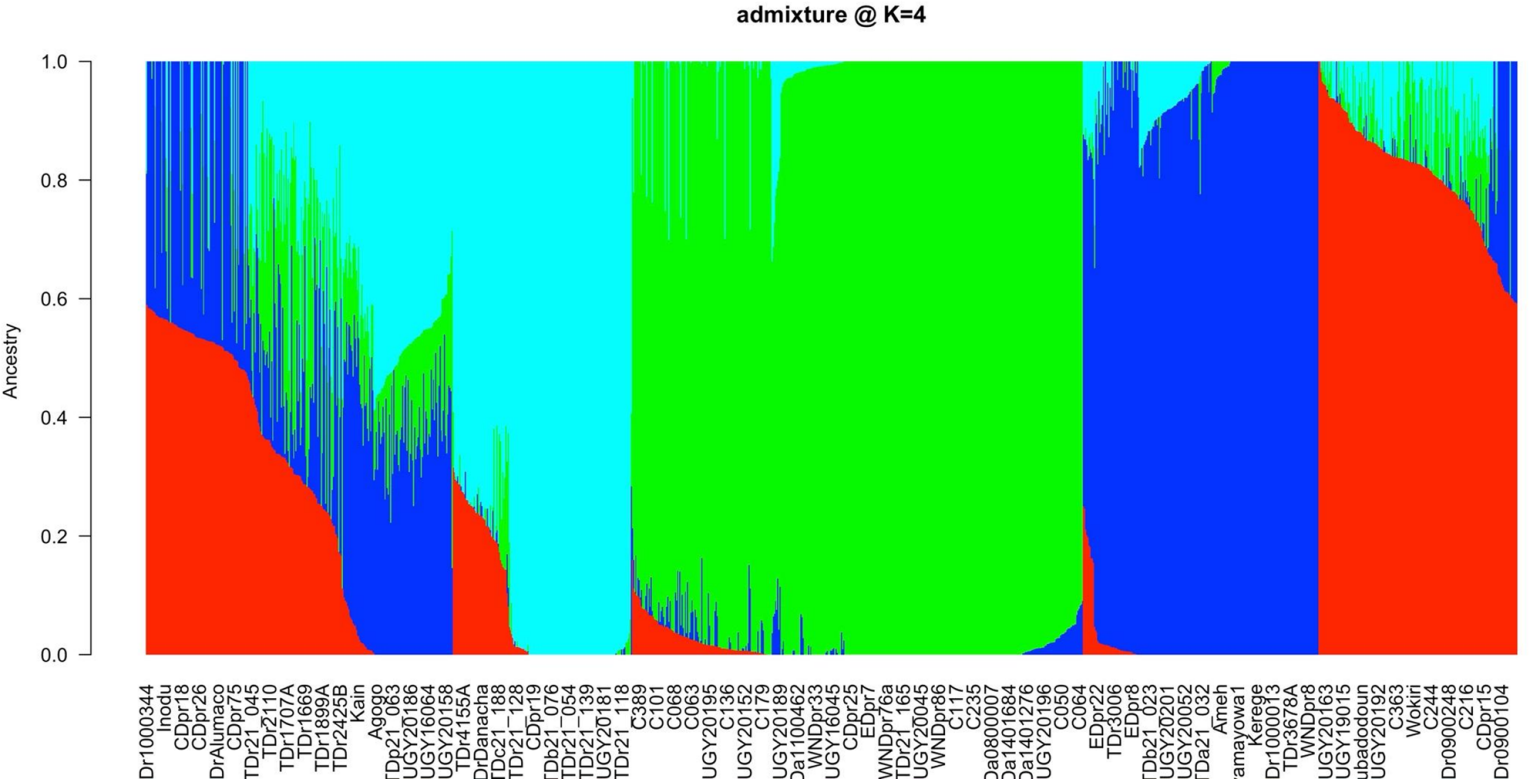
Population structure with K = 4 for 1247 yam accessions into 4 clusters based on the SNP data using Bayesian-based clustering analysis. A vertical bar represents each accession. The coloured sections in a bar indicate the membership coefficients of the accessions in the different clusters. Identified subgroups are: cluster 1 (red), cluster 2 (bleu), cluster 3 (green), and cluster 4 (cyan)

Using the ADMIXTURE, four subpopulations similarly to DAPC were identified (Fig. 5), A total of 968 genotypes were successfully assigned to four subpopulations with an ancestry probability ≥ 70%. The remaining 22.4% (279 genotypes) were classified as admixed withancestry probability < 70%. In group 1 (red), majority of the yam genotypes were.

*D. rotundata* collected from different countries. Group 2 (blue) included 214 accessions, composed of *D. rotundata* (115), with some *D. cayenensis* [6], and *D. praehensilis* (63). Group 3 (green) contained mainly genotypes of *D. alata* from Côte d’Ivoire. while Group 4 (cyan) had a total of 163 accessions of *D. rotundata* (105) and *D. praehensilis* [33]. The admixture accessions were breeding lines of both *D. rotundata* and *D. alata* with few accessions of *D. praehensilis.*

**Fig. 6:**
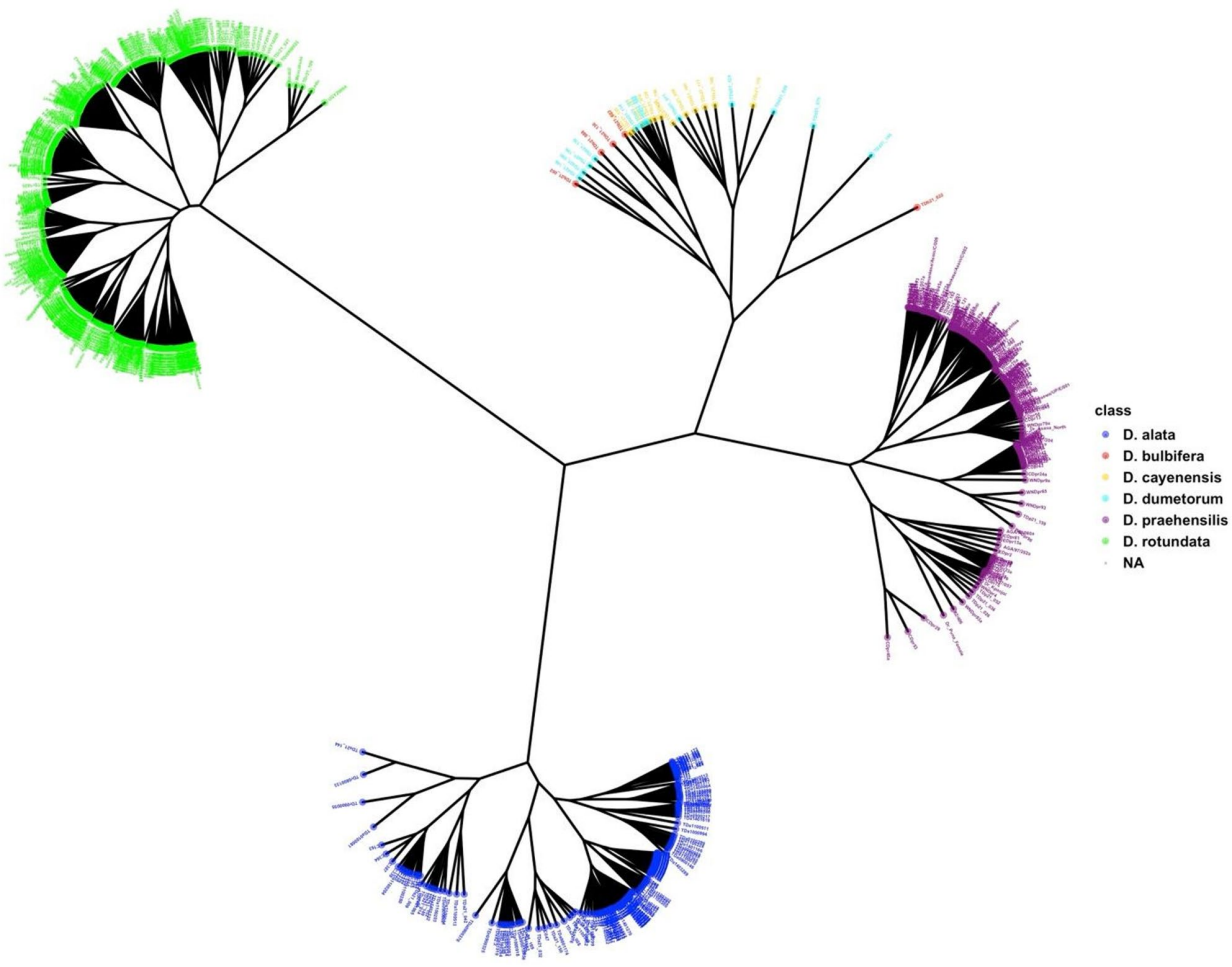
Unrooted Hierarchical cluster based on Jaccard genetic dissimilarity matrix of 1247 showing the grouping pattern of the Dioscorea species accessions, independent of the origin country

To assess the evolutionary relationships among *Dioscorea* species, we constructed a Neighbor-Joining (NJ) phylogenetic tree based on genome-wide SNP data (Fig. 6). The tree clearly differentiates the accessions by species, with well-resolved clusters corresponding to *D. rotundata*,* D. alata*,* D. praehensilis*,* D. bulbifera*,* D. dumetorum*,* and D. cayenensis*. *Dioscorea rotundata* accessions (green) form a highly diverse clade, indicating moderate genetic diversity among both landraces and breeding lines. In contrast, *D. alata* (blue) also forms a distinct clade, though with slightly low internal diversity and several extended branches.

A third major cluster comprises *D. praehensilis* (purple), which shows a wide and deeply branched clade, suggesting substantial intra-specific genetic variation.

The remaining species *D. cayenensis* (orange), *D. bulbifera* (red), and *D. dumetorum* (cyan) occupy more peripheral positions on the tree and are clearly separated from the core clusters, reflecting their more divergent genomic backgrounds and supporting their classification as genetically distinct species within the genus.

Overall, the NJ tree topology supports the species classification derived from previous PCA and structure analyses and provides additional evidence of strong species-level divergence, particularly between cultivated and wild/semi-domesticated yam relatives.

**Fig. 7:**
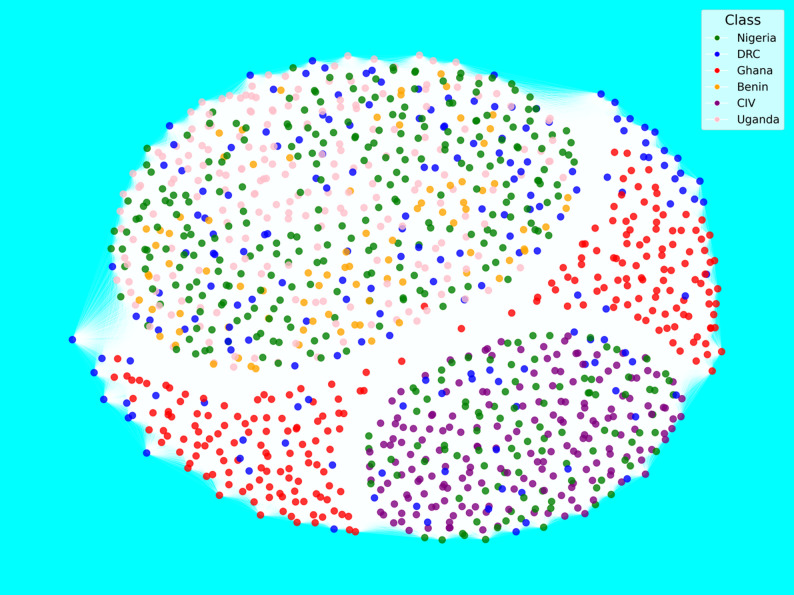
Network-based visualization of genetic relatedness among yam genotypes from Nigeria, Ghana, Benin, Côte d’Ivoire, the Democratic Republic of Congo, and Uganda

**Fig. 8:**
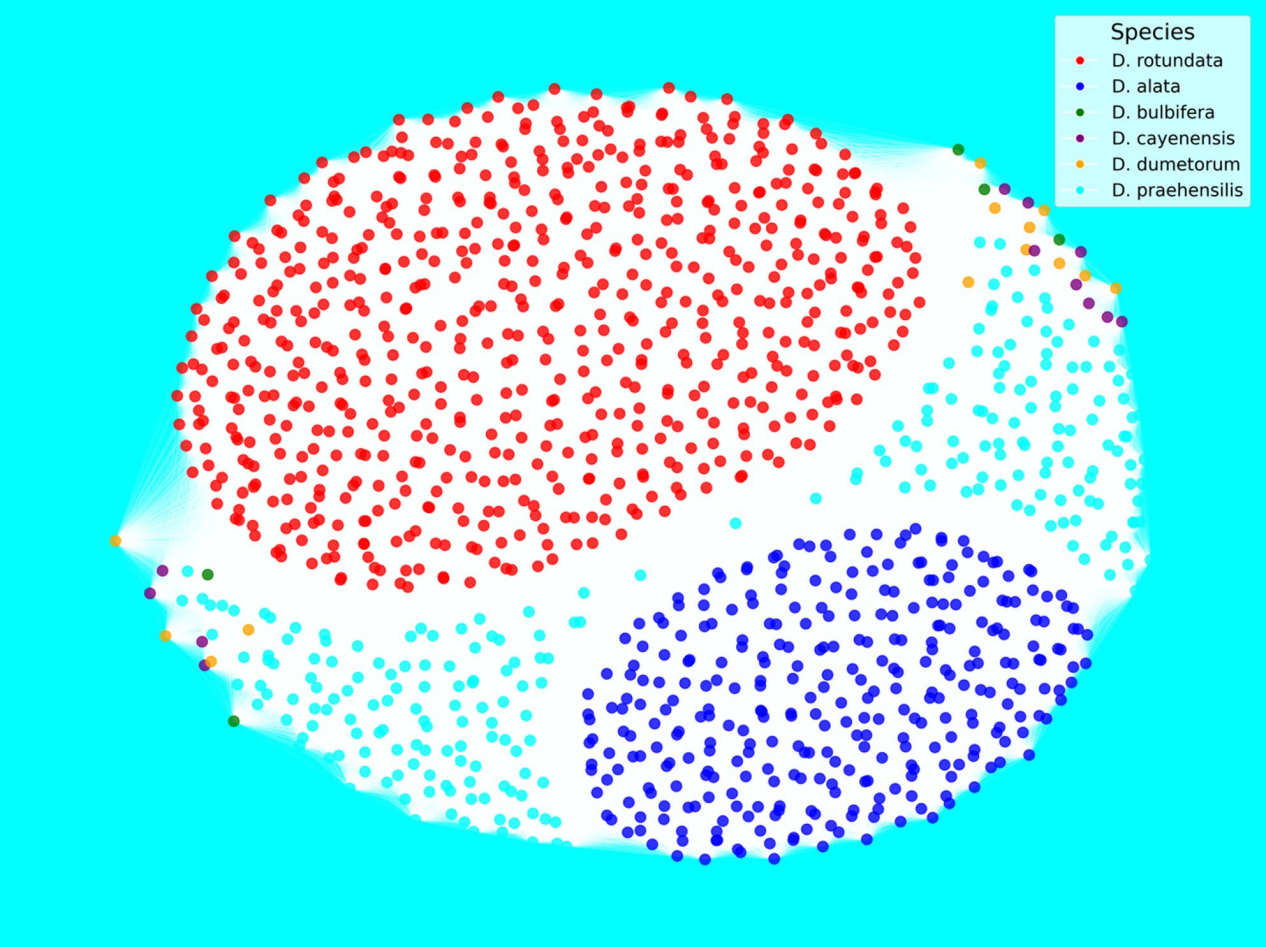
Network-based visualization of genetic relatedness among the six yam species

To gain deeper insights into the genetic relationships among yam accessions, we conducted a network analysis based on pairwise genetic distances and visualized the results using force-directed graphs. Two complementary network views were generated: one representing the country of origin (Fig. 7) and the other species identity (Fig. 8). In both networks, nodes represent individual genotypes, and edges denote genetic similarity above a defined threshold.

Figure 7 displays the network topology with nodes color-coded according to geographic origin, including Nigeria, Ghana, Benin, Côte d’Ivoire, DRC, and Uganda. The network structure reveals extensive interconnections across accessions from different countries, suggesting gene flow or the sharing of germplasm across national borders. A moderately cohesive subnetwork is observed toward the lower right of the graph, largely composed of accessions from Benin and Uganda, suggesting some country-specific structure. However, a broader, more diffuse cloud in the upper half of the network contains a mix of accessions from Ghana, Nigeria, Côte d’Ivoire, and DRC, indicating high genetic similarity among these groups.

In contrast, Fig. 8 shows the same network with nodes colored by species: *D. rotundata*, *D. alata*,* D. praehensilis*,* D. cayenensis*,* D. bulbifera*, and *D. dumetorum*. Here, a much clearer structuring pattern emerges. Three major clusters dominate the graph: a large, tightly connected cluster of *Dioscorea rotundata* accessions (red nodes); a second cluster containing mostly *Dioscorea alata* (blue) and *Dioscorea praehensilis* (cyan); and a sparse periphery consisting of more genetically distant species such as *Dioscorea cayenensis*,* Dioscorea dumetorum*, and *Dioscorea bulbifera*. This species-based organization aligns well with the ADMIXTURE, DAPC, and phylogeny tree and confirms that species identity, rather than geography, is the primary driver of genetic structure in the yam diversity panel analyzed.

**Fig. 9:**
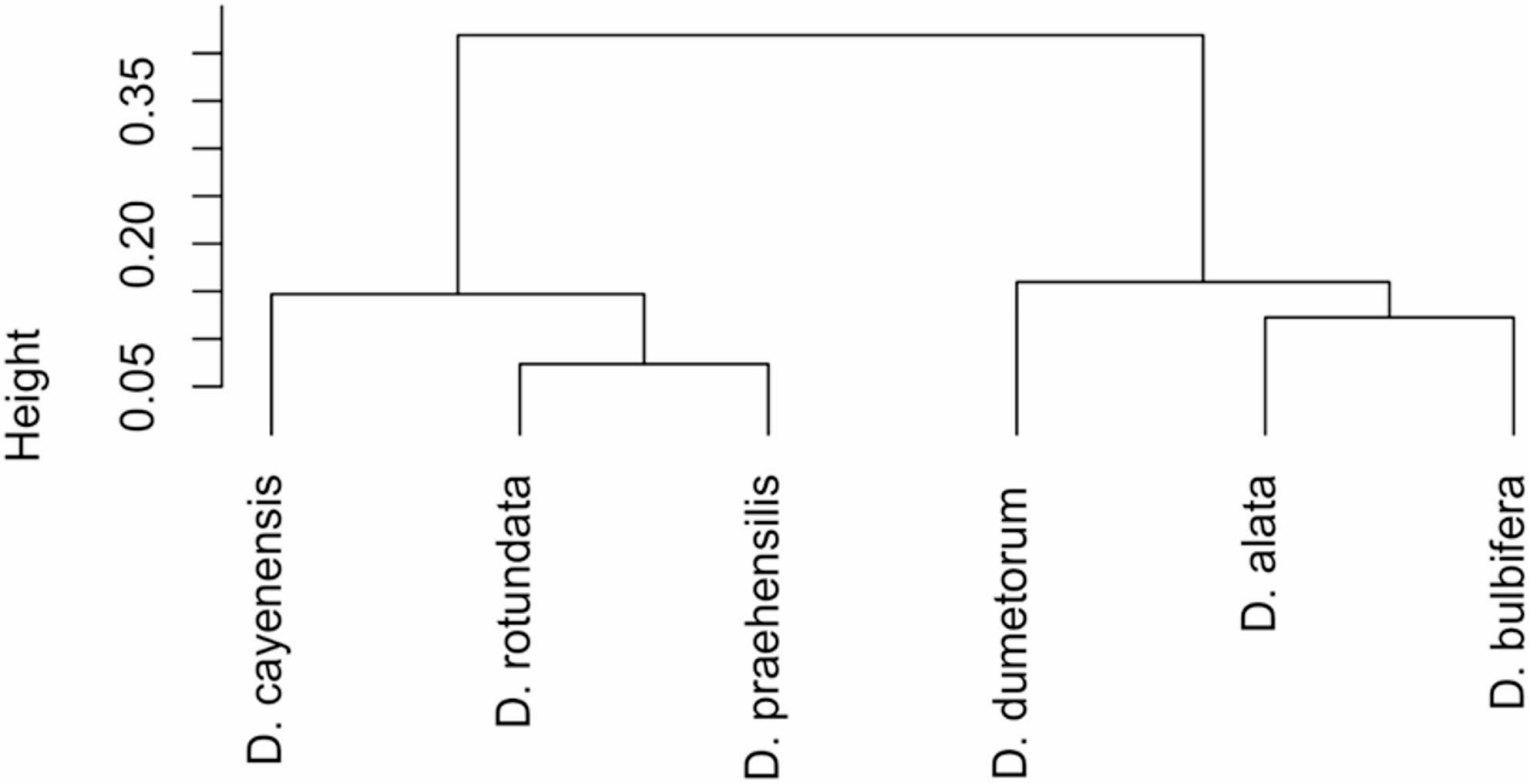
Hierarchical Dendogram-based on Dioscorea species evolutionary (D is for Dioscorea)

The AMOVA results provided further insights into genetic differentiation across countries and species (Tables 4 and 5). Among-country, an average fixation index (Fst) of 0.07, indicating moderate differentiation (Table 4). The closest genetic relationship was between Ghana and Nigeria (Fst = 0.08) (Table 4), while the highest genetic distance was observed between Uganda and DRC (Fst = 0.16) (Table 4). Within-species variation showed moderate differentiation (average Fst = 0.14) (Table 5), with the highest variation observed in *Dioscorea rotundata* (Fst = 0.18) and the lowest in *D. cayenensis* (Fst = 0.04) (Table 5). Between-species Fst values ranged from 0.10 to 0.28, with the closest relationship was between *D. praehensilis* and *D. cayenensis* (Fst = 0.10), and the highest divergence was between *D. rotundata* and *D. alata* (Fst = 0.28). Cluster analysis grouped the six yam species into two major clusters, highlighting the genetic proximity of *D. rotundata* and *D. praehensilis* and the distinctiveness of *D. dumetorum* (Fig. 9).

**Table 4:** Fixation index (Fst) based on countries of origin

Country	Benin	CIV	DRC	Ghana	Nigeria	Uganda
Benin	0.02					
CIV	0.1	0.04				
DRC	0.05	0.06	0.07			
Ghana	0.06	0.15	0.11	0.06		
Nigeria	0.06	0.08	0.09	0.08	0.08	
Uganda	0.04	0.13	0.16	0.03	0.07	0.01
Average	0.055	0.092	0.108	0.057	0.075	0.01

The diagonal value represent the diversity index within country. DRC, Democratic Republic of Congo; CIV, Côte d’Ivoire.

**Table 5:** Fixation index (Fst) based on Yam species

Species	TDr	TDa	TDc	TDp	TDd	TDb
TDr	0.18					
TDa	0.28	0.12				
TDc	0.12	0.22	0.04			
TDp	0.15	0.27	0.10	0.14		
TDd	0.22	0.19	0.23	0.21	0.08	
TDb	0.21	0.11	0.30	0.17	0.11	0.02
Average	0.193	0.182	0.168	0.173	0.095	0.02

TDr, *Dioscorea rotundata*; TDa, *D. alata*; TDc, *D. cayenensis*; TDp, *D. praehensilis;* TDd, *D. dumetorum;*

TDb, *D. bulbifera*

## Discussion

This study presents one of the most comprehensive assessments of genetic diversity in yam (*Dioscorea spp*.) conducted in sub-Saharan Africa, comprising 1,247 accessions across six countries and six yam species, including both cultivated and wild related. Using 7,648 (https://figshare.com/account/items/29314031/edit*)* SNP markers, we generated a robust genomic framework to elucidate the evolutionary relationships, population structure, and genetic differentiation within African yam germplasm.

Diversity estimates based on SNP data revealed moderate polymorphism levels across species. The average observed heterozygosity (Ho = 0.259) was consistently lower than the expected heterozygosity (He = 0.441), suggesting a degree of excess homozygosity. This is consistent with the clonally propagated nature of yam, limited recombination, and potential sub-structuring within population patterns previously reported in clonally propagated crops [12, 19, 44]. The slightly higher observed heterozygosity (Ho = 0.288) for *D. bulbifera* in this study compared with the value reported by [23] (Ho = 0.262) could be attributed to differences in germplasm composition, marker density, and population structure. The inclusion of accessions from multiple geographic regions and the use of high-resolution SNP markers may have contributed to the broader allelic representation and increased heterozygosity observed. Expected heterozygosity (He) ranged from 0.262 to 0.277, with *D. cayenensis* showing the highest value (He = 0.454), indicating species-specific variation in allele richness and genetic diversity [18, 45, 46].

These diversity estimates are in agreement with previous reports based on SSR and SNP marker analyses [7, 17, 19]. The observed stability in MAF (0.236) and PIC (0.360), with the Nigerian population and *D. rotundata* exhibiting the highest PIC values (0.246 and 0.363, respectively), underscores the high informativeness and discriminatory power of the SNP panel. This further highlights its suitability for genetic diversity assessment and breeding applications in Dioscorea species [24]. The moderate disparity between expected (He) and observed (Ho) heterozygosity suggests a reduction in genetic variability within the population. This pattern may reflect historical bottlenecks, domestication pressures, and allele fixation events associated with clonal propagation and recurrent selection processes previously reported during the domestication and genetic refinement of Dioscorea rotundata [12, 13, 47].

Population structure analysis identified four well-resolved genetic clusters using ADMIXTURE and DAPC, with a proportion of admixed individuals indicative of historical gene flow and interspecific introgression [24, 38]. These patterns are consistent with traditional farmer practices involving clonal propagation and exchange of diverse landraces, which promote population connectivity [48]. Phylogenetic overlap between *D. rotundata* and *D. praehensilis* supports the hypothesisof *D. rotundata* been originated from a long domestication process from wild progenitors.Scarcelli et al., (2006) and Sugihara et al., (2020) highlighted the value of *D. praehensilis* as a reservoir of favorable alleles for traits such as disease resistance and yield potential [13, 21].

In contrast, *Dioscorea alata* showed relatively low intra-species variability, likely due to its introduction from the Pacific with a narrow founder base, followed by clonal expansion and limited recombination in Africa [49, 50]. This supports the need to broaden its genetic base through introgression and targeted diversity enrichment.

AMOVA revealed moderate differentiation among countries (Fst = 0.07) and higher differentiation among species (Fst = 0.14), in agreement with [38, 51]. The highest interspecific divergence (Fst = 0.28) between *D. rotundata* and *D. alata* reflects their divergent origins, while the lowest Fst (0.10) between *D. praehensilis* and *D. cayenensis* may suggest shared ancestry or convergent evolution under similar selection pressures. These findings reinforce the importance of conserving both wild and cultivated species to ensure long-term breeding gains [2].

From a breeding perspective, the distinct genetic clusters and country-level stratification provide a valuable framework for parental line selection. Moreover, this study demonstrates the utility of DArTseq and whole-genome resequencing platforms for high-resolution diversity analysis in yam, a crop often challenged by its polyploidy and clonal nature [52]. The consistency across ADMIXTURE, DAPC, and phylogenetic clustering confirms the reliability of the dataset and its suitability for core collection development and pre-breeding pipelines.

## Conclusion

In this study, we generated an informative dataset across Africa to reveal the genetic diversity of yam. The study offers important insights into the genetic architecture and population dynamics of yam in Africa. The identified diversity patterns and population structure form a valuable foundation for breeding strategies, germplasm conservation, and trait mapping. Considering the complex domestication history, widespread informal seed systems, and broad agroecological distribution of yam, future research should prioritize the inclusion of geographically representative and genetically diverse accessions. Such efforts will strengthen genomic-assisted breeding, enhance trait discovery, and ensure the long-term conservation and utilization of Africa’s yam genetic resources.

## Data Availability

Data used in this study is available through the following website and can freely be downloaded. [https://figshare.com/account/items/29314031/edit](https:/figshare.com/account/items/29314031/edit).

## Abbreviations

SNP: Single nucleotide polymorphism
MAF: Minor allele frequency
Ho: Observed heterozygosity
He: Expected heterozygosity
PIC: Polymorphic information content
FST: Fixation index
AMOVA: Analysis of molecular variance
IBS: Identity-by-state
NJ: Neighbor-joining
PCA: Principal component analysis
DAPC: Discriminant analysis of principal components
ADMIXTURE: Model-based clustering algorithm for estimating ancestry proportions
CV: Cross-validation
BIC: Bayesian information criterion
LD: Linkage disequilibrium
HWE: Hardy–Weinberg equilibrium
r²: Squared correlation coefficient
K: Number of clusters
DOI: Digital object identifier
SD: Standard deviation
SE: Standard error
CI: Confidence interval
IQR: Interquartile range

## References

[1] Orkwor C, Asiedu R, Ekanayake IJ. Food yams advances in research. Adv Res. 1998;3(2):30–62.

[2] Asiedu R, Sartie A. Crops that feed the world 1. Yams. Food Secur. 2010;2(4):305–15.

[3] Alabi TR, Adebola PO, Asfaw A, De Koeyer D, Lopez-Montes A, Asiedu R. Spatial multivariate cluster analysis for defining target population of environments in West Africa for Yam breeding. Int J Appl Geospatial Res. 2019;10(3):1–30.

[4] Edem ID, Nkereuwem ME. Crucial Roles of Tuber Crops and the Development Activities in the Global Food System. Am J Agric Sci. 2015;2(2):42–9 http://www.aascit.org/journal/ajas.

[5] Kulasinghe WMAA, Ranaweera KKTN. Physical, chemical and biological aspects of < em > Dioscorea yams and potential value additions. J Agric Value Addit. 2019;2(1):52–61.

[6] Degla P, Sourokou N, Food. Socio-Cultural and economic importance of Yam in the North-East of Benin. J Agric Environ Sci. 2020;9(2):18–25.

[7] Mignouna HD, Abang MM, Aiedu R, Geeta R. Yam (*Dioscorea*) husbandry: cultivating Yams in the field or greenhouse. Cold Spring Harb Protoc. 2009;4(11):2–4. 10.1101/pdb.prot5324 20150063

[8] Mawoneke KG, Kwiri R, Ndemera M. A concise review of yam (*Dioscorea* spp.) starch: extraction, chemical composition, physicochemical properties and its potential food applications. Cogent Food Agric [Internet]. 2025;11(1). Available from: 10.1080/23311932.2024.2447900

[9] NORMAN MJT. C. J. PEARSON PGES. Physiological ecology of tropical plants: Second edition. In: Physiological Ecology of Tropical Plants: Second Edition. 2008:23(3):1–458.

[10] Agre P, Asibe F, Darkwa K, Edemodu A, Bauchet G, Asiedu R, et al. Phenotypic and molecular assessment of genetic structure and diversity in a panel of winged Yam (*Dioscorea alata*) clones and cultivars. Sci Rep. 2019;9(1):1–11. 31796820 10.1038/s41598-019-54761-3 PMC6890776

[11] Sugihara Y, Kudoh A, Oli MT, Takagi H, Natsume S, Shimizu M, et al. Population Genomics of Yams: Evolution and Domestication of Dioscorea Species. Crop Plant. 2021;4(3):837–64.

[12] Scarcelli N, Cubry P, Akakpo R, Thuillet AC, Obidiegwu J, Baco MN, et al. Yam genomics supports West Africa as a major cradle of crop domestication. Sci Adv. 2019;5(5):1–7. 10.1126/sciadv.aaw1947 PMC6527260 31114806

[13] Sugihara Y, Darkwa K, Yaegashi H, Natsume S, Shimizu M, Abe A, et al. Genome analyses reveal the hybrid origin of the staple crop white Guinea Yam (*Dioscorea rotundata*). Proc Natl Acad Sci U S A. 2020;117(50):31987–92. 33268496 10.1073/pnas.2015830117 PMC7749330

[14] Worojie TB, Asfaw BT, Mengesha WA. Cultivation and possible domestication of feral and possibly wild yams (*Dioscorea* spp.) in Southwest Ethiopia: ethnobotanical and morphological evidence. Plant Signal Behav [Internet]. 2021;16(5). Available from: 10.1080/15592324.2021.1879531 10.1080/15592324.2021.1879531 PMC8078503 33678151

[15] Cao T, Sun J, Shan N, Chen X, Wang P, Zhu Q, et al. Uncovering the genetic diversity of Yams (*Dioscorea* spp.) in China by combining phenotypic trait and molecular marker analyses. Ecol Evol. 2021;11(15):9970–86. 34367553 10.1002/ece3.7727 PMC8328405

[16] Hu X, Wang X, Xu A, Guan W, Han L, Zhang P. Population structure and genetic diversity of Chinese yams based on SNP molecular markers. Genet Resour Crop Evol [Internet]. 2025;72(7):8469–88. Available from: 10.1007/s10722-025-02468-y

[17] Tamiru M, Becker HC, Maass BL. Comparative analysis of morphological and farmers’ cognitive diversity in Yam landraces (*Dioscorea* spp.) from Southern Ethiopia. Trop Agric Dev. 2011;5(1):28–43.

[18] Amponsah Adjei E, Esuma W, Alicai T, Bhattacharjee R, Dramadri IO, Edema R, et al. Genetic diversity and population structure of uganda’s Yam (*Dioscorea* spp.) genetic resource based on DArTseq. PLoS ONE. 2023;18(2):18–29. 10.1371/journal.pone.0277537 PMC9928066 36787288

[19] Agre PA, Dassou AG, Loko LEY, Idossou R, Dadonougbo E, Gbaguidi A, et al. Diversity of white Guinea yam (*Dioscorea rotundata Poir.*) cultivars from Benin as revealed by agro-morphological traits and SNP markers. Plant Genet Resour Characterisation Util. 2021;19(5):437–46.

[20] Mekbib F, Hussein S, Gebre E, Agricultural J, Africa S, Ababa A. Genetic Variability, correlation and path analysis on the storage tuber yield and yield components of Yam (*Dioscorea* spp.) from Southwest Ethiopia. Genet Resour Crop Evol. 2003;4(1):98–118.

[21] Tostain S, Agbangla C, Scarcelli N, Mariac C, Daïnou O, Berthaud J, et al. Genetic diversity analysis of yam cultivars (*Dioscorea rotundata Poir.*) in Benin using simple sequence repeat (SSR) markers. Plant Genet Resour Characterisation Util. 2007;5(2):71–81.

[22] Tamiru M, Becker HC, Maass BL. Genetic diversity in Yam germplasm from Ethiopia and their relatedness to the main cultivated Dioscorea species assessed by AFLP markers. Crop Sci. 2007;47(4):1744–53.

[23] Ekaette E, Nwofia E, Okocha P, Nnnabue I, Eluwa K, Obidiegwu J, et al. Exploring the genetic diversity and population structure of aerial yams (*Dioscorea bulbifera* L.) DArT-seq and agronomic traits. PLoS One. 2024;19(8):1–19. 10.1371/journal.pone.0306631. 10.1371/journal.pone.0306631 PMC11343425 39178185

[24] Darkwa K, Agre P, Olasanmi B, Iseki K, Matsumoto R, Powell A, et al. Comparative assessment of genetic diversity matrices and clustering methods in white Guinea yam (*Dioscorea rotundata*) based on morphological and molecular markers. Sci Rep. 2020;10(1):1–14. 10.1038/s41598-020-69925-9. 32764649 10.1038/s41598-020-69925-9 PMC7413250

[25] Diouf MB, Festus R, Silva G, Guyader S, Umber M, Seal S, et al. Viruses of Yams (*Dioscorea* spp.): current gaps in knowledge and future research directions to improve disease management. Viruses. 2022;14(9):1–23. 10.3390/v14091884 PMC9501508 36146691

[26] Epping J, Laibach N. An underutilized orphan tuber crop—Chinese yam: a review. Planta [Internet]. 2020;252(4):1–19. Available from: 10.1007/s00425-020-03458-3 10.1007/s00425-020-03458-3 PMC7505826 32959173

[27] Ignatius NA, Rachid H, Pierre NS, Achiangia PN. Yam (*Dioscorea* spp.) production trends in cameroon: A review. Afr J Agric Res. 2019;14(26):1097–110.

[28] Matthew J, Faluyi JO. Chromosomal analysis of eight cultivars in three species of cultivated Yam (*Dioscorea* L.) species in Nigeria. Caryologia. 2021;74(2):3–9.

[29] Faluyi JO, Matthew JO, Collection. Characterizaton and Conservation of Genetic Resources of Yam Cultivars From Ekiti. Genet Resour Crop Evol. 2021;2(3):12–20.

[30] Asfaw BT, Worojie TB, Mengesha WA. Assessing morphological diversity in Ethiopian yams (*Dioscorea* spp.) and its correspondence with folk taxonomy. Syst Biodivers [Internet]. 2021;19(5):471–87. Available from: 10.1080/14772000.2021.1890269

[31] Worojie TB, Asfaw BT, Mengesha WA. Indigenous biosystematics of Yams (*Dioscorea* spp.) in Southwest ethiopia: folk taxonomy, ethnolinguistic analysis, and folk descriptors. J Ethnobiol Ethnomed. 2021;17(1):1–15. 33386077 10.1186/s13002-020-00427-8 PMC7777353

[32] Muthamia ZK, Nyende AB, Mamati EG, Ferguson ME, Wasilwa J. Determination of ploidy among Yam (*Dioscorea* spp.) landraces in Kenya by flow cytometry. Afr J Biotechnol. 2014;13(3):394–402.

[33] Adejumobi II, Agre PA, Onautshu DO, Adheka JG, Cipriano IM, Monzenga JCL, et al. Assessment of the Yam landraces (*Dioscorea* spp.) of DR congo for reactions to pathological Diseases, yield Potential, and tuber quality characteristics. Agric. 2022;12(5):14–21.

[34] Mondo JM, Agre PA, Edemodu A, Adebola P, Asiedu R, Akoroda MO, et al. Floral biology and pollination efficiency in Yam (*Dioscorea* spp). Agric. 2020;10(11):1–21.

[35] Agre PA, Edemodu A, Obidiegwu JE, Adebola P, Asiedu R, Asfaw A. Variability and genetic merits of white Guinea Yam landraces in Nigeria. Front Plant Sci. 2023;14(February):1–12. 10.3389/fpls.2023.1051840 PMC9940711 36814760

[36] Bakayoko L, N’Da Pokou D, Kouassi AB, Agre PA, Kouakou AM, Dibi KEB, et al. Diversity of water Yam (*Dioscorea Alata* L.) accessions from côte d’ivoire based on Snp markers and agronomic traits. Plants. 2021;10(12):1–18. 10.3390/plants10122562 PMC8705775 34961033

[37] Adewumi AS, Asare PA, Akintayo OT, Adejumobi II, Adu MO, Taah KJ et al. Genetic architecture of post-harvest tuber quality traits in Bush Yam (*Dioscorea praehensilis Benth.*) germplasm through association mapping. Front Hortic. 2024;3(4):24–32.

[38] Adejumobi II, Agre PA, Adewumi AS, Shonde TE, Cipriano IM, Komoy JL, et al. Association mapping in multiple Yam species (*Dioscorea* spp.) of quantitative trait loci for yield-related traits. BMC Plant Biol. 2023;23(1):1–16. 37434107 10.1186/s12870-023-04350-4 PMC10334582

[39] Adewumi AS, Agre PA, Asare PA, Adu MO, Taah KJ, Mondo JM, et al. Exploring the Bush Yam (*Dioscorea praehensilis benth*) as a source of agronomic and quality trait genes in white Guinea Yam (dioscorea rotundata poir) breeding. Agronomy. 2022;12(1):1–16.

[40] Purcell S, Neale B, Todd-Brown K, Thomas L, Ferreira MAR, Bender D, et al. PLINK: A tool set for whole-genome association and population-based linkage analyses. Am J Hum Genet. 2007;81(3):559–75. 17701901 10.1086/519795 PMC1950838

[41] Jombart T, Adegenet. A R package for the multivariate analysis of genetic markers. Bioinformatics. 2008;24(11):1403–5. 18397895 10.1093/bioinformatics/btn129

[42] Paradis E, Claude J, Strimmer K, APE. Analyses of phylogenetics and evolution in R Language. Bioinformatics. 2004;20(2):289–90. 14734327 10.1093/bioinformatics/btg412

[43] Hagberg AA, Schult DA, Swart PJ. Exploring network Structure, Dynamics, and function using networkX. Proc 7th Python Sci Conf. 2008;2(3):11–5.

[44] Arnau G, Nemorin A, Maledon E, Abraham K. Revision of ploidy status of Dioscorea Alata L. (Dioscoreaceae) by cytogenetic and microsatellite segregation analysis. Theor Appl Genet. 2009;118(7):1239–49. 19253018 10.1007/s00122-009-0977-6

[45] Castañeda-Cardona CC, Morillo-Coronado Y, Morillo AC. Assessing the genetic diversity of Dioscorea Alata and related species from Colombia through inter-simple sequence repeat (Issr) markers. Chil J Agric Res. 2020;80(4):608–16.

[46] Korsa F, Feyissa T, Dessalegn O, Mekonnen T. Genetic diversity and population structure of Yam (*Dioscorea species*) from Western Ethiopia as revealed by simple sequence repeat markers. 2022;1–19. Available from: https://www.researchsquare.com/article/rs-1905131/v1

[47] Tamiru M, Natsume S, Takagi H, White B, Yaegashi H, Shimizu M, et al. Genome sequencing of the staple food crop white Guinea Yam enables the development of a molecular marker for sex determination. BMC Biol. 2017;15(1):1–20. 28927400 10.1186/s12915-017-0419-x PMC5604175

[48] Chaïr H, Cornet D, Deu M, Baco MN, Agbangla A, Duval MF, et al. Impact of farmer selection on Yam genetic diversity. Conserv Genet. 2010;11(6):2255–65.

[49] Mignouna HD. Yam (*Dioscorea ssp*.) domestication by the Nago and Fon ethnic groups in Benin. Entomol Exp Appl. 2003;103(3):239–48.

[50] Egesi CN, Asiedu R, Ude G, Ogunyemi S, Egunjobi JK. AFLP marker diversity in water Yam (*Dioscorea Alata L*). Plant Genet Resour. 2006;4(3):181–7.

[51] Bhattacharjee R, Agre P, Bauchet G, Koeyer D, De, Lopez-montes A, et al. Genotyping-by-Sequencing to Unlock Genetic Diversity and Population Structure in White Yam. MDPI Agron. 2020;4(3):16-27.

[52] Owiti AA, Bargul JL, Obiero GO, Nyaboga EN. Analysis of genetic diversity and population structure in Yam (*Dioscorea Species*) germplasm using start codon targeted (SCoT) molecular markers. Int J Plant Biol. 2023;14(1):299–311.

